# Integrative multi-omic profiling of the neoantigen landscape of glioblastoma for the development of therapeutic vaccines reveals vast heterogeneity in immunogenic signatures

**DOI:** 10.3389/fonc.2025.1507632

**Published:** 2025-03-21

**Authors:** Qingtang Lin, Yukui Wei, Geng Xu, Leiming Wang, Feng Ling, Xiaojie Chen, Ye Cheng, Yiming Zhou

**Affiliations:** ^1^ Department of Neurosurgery, Brain Tumor and Skull-Base Center, Xuanwu Hospital, Capital Medical University, Beijing, China; ^2^ Department of Pathology, Xuanwu Hospital, Capital Medical University, Beijing, China; ^3^ Base&Byte Biotechnology Co., Ltd, Beijing, China

**Keywords:** glioblastoma, neoantigen, sequencing, heterogeneity, immunotherapy

## Abstract

**Introduction:**

Glioblastoma (GBM) is the most common primary brain malignancy. Few neoantigens have been tested in trials as the cancer vaccine against GBM.

**Methods:**

To better understand the neoantigen landscape and its associated tumor microenvironment (TME) for the optimized vaccine design of our initiated GBM trial, we apply the integrative multi-omics approach to comprehensively profile the mutation, HLA typing, TCR/BCR repertoire, immune cell components on the tumor tissue and peripheral blood mononuclear cell (PMBC) specimen of 24 GBM patients.

**Results:**

On average, 148 mutated genes and 200 mutated sites per patient were identified, with no predominant mutated sites and genes in this cohort. Diversified HLA genotypes and expression rate across A, B, and C alleles, with A30:01&A11:01, B13:02, and C06:02, as the most frequent genotypes at respective alleles. Clustered CDR3 of TCR/BCR existed in tumor tissue with decreased richness compared with PMBC. NK and Th1 cells were revealed as the predominant immune cells within the tumor microenvironment (TME). Neoantigens were feasible predicted and designed for each patient, with an average number of 107. Very few neoantigens were shared by more than two patients and no dominant neoantigen could be identified. A minimum of 11-peptide bulk was required to cover this 24-patient cohort, guaranteeing each patient could have at least one neoantigen.

**Discussion:**

In summary, our data reveals a heterogeneous landscape of the neoantigen and its associated immune TME of GBM, based on which a peptide bulk is feasibly developed to cover these patients as a cohort.

## Introduction

Glioblastoma (GBM)is the most common primary brain malignancy, accounting for nearly 25% of all primary brain tumors (1, 2). The prognosis for patients with GBM remains poor, with median overall survival ranging from 14.6 to 20.1 months after the standard therapy consisting of surgery, radiotherapy with concomitant and adjuvant temozolomide chemotherapy, and tumor treating field (3, 4). The major reason for this poor prognosis is largely due to the limited effective therapies (5). Unlike other cancers that have multiple first-line chemotherapeutic drugs, there is only one first-line chemotherapeutic drug, temozolomide (6, 7).

Immunotherapy has changed the practical paradigm for numerous human cancers (8, 9). However, results from previous immunotherapy trials including ours for GBM failed to demonstrate its robust clinical efficacy (10–15). Neoantigens are newly formed antigens resulting from genetic alternations within tumors, such as somatic mutation, alternating RNA splicing, and post-transcriptional modification (16, 17). Exploiting neoantigen as a cancer vaccine against GBM has been attempted, either with a personalized or universal vaccine strategy, demonstrating vaccine-specific immune response and ambiguous clinical efficacy (18–20). The suppressive immune microenvironment of GBM further complexed the lack of efficacy of immunotherapy (21, 22).

To comprehensively profile the neoantigen landscape of GBM and its inherent tumor microenvironment for optimized development of neoantigen-based immunotherapies including our initiated clinical trial (NCT NCT04943718), we applied an integrative multi-omic approach to a cohort of GBM patients. Here, we report our results.

## Materials and methods

### Human tissue samples

Patients with a pathologically confirmed diagnosis of GBM were enrolled in this study. All patients provided written informed consent for sample collection and data analyses. This study was approved by the Ethical Committee of Xuanwu Hospital, Beijing, China.

### Next-generation sequencing

Their tumors were obtained for Whole Exon Sequencing (WES) and RNA-seq experiments, and their peripheral blood was obtained for paired WES experiments and immune repertoire experiments. For detailed methods please refer to **Supplementary Material 1**. The datasets were uploaded and can be found in online repositories. The names of the repository/repositories and accession number(s) can be found below: China National Center for Bioinformation - National Genomics Data Center.

### WES data analysis

#### Sample evaluation

DNA quality was assessed by agarose gel electrophoresis to check for degradation and potential contamination by RNA or proteins. DNA concentrations were quantified precisely using the Qubit fluorometer. Only DNA samples with concentrations ≥0.6 μg were used for library construction.

#### Library construction and capture

Sequencing libraries were constructed using the Agilent SureSelect Human All Exon V6 kit following the manufacturer’s protocol. Genomic DNA was fragmented to 180-280 bp using a Covarissonicator. End repair and A-tailing were performed on the fragmented DNA before ligation to indexed sequencing adapters. The adapter-ligated libraries were pooled and hybridized to biotinylated probes targeting exonic regions. Streptavidin-coated magnetic beads were used to capture probe-bound fragments. Captured exons were amplified by PCR and the final libraries were evaluated for quality control.

#### Library quality control

Initial library quantification was done with Qubit 2.0 fluorometry. Insert sizes were validated on an Agilent 2100 Bioanalyzer to meet target range specifications. Final precise library quantification was performed by qPCR to ensure concentrations ≥3 nM for adequate cluster densities during sequencing.

### Sequencing

Qualified libraries were sequenced on an Illumina NovaSeq 6000 platform to generate 150 bp paired-end reads based on the effective concentrations and desired data yield.

### Data analysis

Paired-end reads were first processed to remove adapters and low-quality sequences using Trimmomatic to get high-quality reads, and then the quality of high-quality reads was assessed using FASTQC (23, 24). High-quality reads were aligned to the Homo Sapiens reference genome (hg19) using BWA (25). Duplicate reads were filtered using Picard Tools (https://broadinstitute.github.io/picard/). The quality of aligned sequence data was assessed using Qualimap 2 (26). Germline variant call was analyzed by GATK HaplotypeCaller (27). Somatic variants were identified by Mutect2 and FilterMutectCalls methods in GATK software[https://www.biorxiv.org/content/10.1101/861054v1]. Mutation landscapes were analyzed by Maftools (28).

### RNA-seq data analysis

#### Sample collection and preparation

Sequencing libraries were constructed from 250 ng of purified PCR2 products using the TIANSeq Fast DNA Library Kit (TIANGEN) with TIANSeq Single-Indexed Adapters (Illumina). End repair, A-tailing, and adapter ligation were performed following the manufacturer’s protocol. Adapter-ligated products were purified with the DNA Clean-up Kit (CWBIO) and further amplified by PCR. Final libraries were purified by an additional DNA Clean-up Kit step and size-selected for 400-800 bp fragments using AMPure XP beads. Libraries were quantified by Qubit fluorometry and the insert size distribution was analyzed on an Agilent 2100 Bioanalyzer. Libraries with the expected insert size were accurately quantified by qRT-PCR to ensure the final concentration was greater than 2 nM. Total RNA was extracted from samples using TRIzol reagent according to the manufacturer’s instructions. RNA degradation and contamination were monitored by agarose gel electrophoresis. RNA purity was assessed by spectrophotometry using a NanoPhotometer spectrophotometer (IMPLEN). RNA integrity was evaluated using an Agilent 2100 Bioanalyzer (Agilent Technologies).

#### Library preparation for transcriptome sequencing

Sequencing libraries were prepared from 1 μg of total RNA per sample using the NEBNext Ultra RNA Library Prep Kit for Illumina (NEB) following the manufacturer’s protocol. Briefly, mRNA was enriched using oligo-dT magnetic beads. Fragmentation was carried out in NEBNext First Strand Synthesis Reaction Buffer. First-strand cDNA synthesis was performed using random hexamer primers and M-MuLV Reverse Transcriptase (RNase H-). Second strand cDNA was subsequently synthesized using DNA Polymerase I and RNase H. Overhangs were converted into blunt ends and adenylated at 3’ ends. NEBNext adaptors with hairpin loop structures were ligated for hybridization. cDNA fragments of 250-300 bp were size-selected using AMPure XP beads (Beckman Coulter). Before PCR amplification, 3 μl of USER enzyme (NEB) was added to enzymatically digest the uracil-containing adaptors. PCR was performed using Phusion High-Fidelity DNA polymerase, Universal PCR primers, and index primers. AMPure XP beads were used to purify the final PCR products. Library quality was assessed on an Agilent 2100 Bioanalyzer.

Index-coded libraries were clustered on a cBot Cluster Generation System using a TruSeq PE Cluster Kit v3-cBot-HS (Illumina). Clustered libraries were then sequenced on an Illumina Novaseq platform to generate 150 bp paired-end reads.

### Data pre-processing

Raw sequencing reads in fastq format were processed using in-house Perl scripts to remove adapter sequences, poly-N-containing reads, and low-quality reads. The processed clean reads were used for subsequent analyses. Quality control metrics including Q20, Q30, and GC content were calculated for the clean data.

### Data analysis

Paired-end reads were first processed to remove adapters and low-quality sequences using Trimmomati to get high-quality reads, and then the quality of high-quality reads was assessed using FASTQC (23, 24). High-quality reads were aligned using HISAT2 (29). The quality of aligned sequence data was assessed using Qualimap 2 (26). Normalized gene-level expression measurements were calculated as transcripts per million (TPM) with StringTie (30).

### Immune repertoire data analysis

#### Sample collection and preparation

RNA was extracted following the manufacturer’s protocol. RNA purity was assessed by spectrophotometry (OD260/280 and OD260/230 ratios) using a NanoDrop ultra-micro spectrophotometer.

#### Library construction and quality control

##### cDNA Synthesis

cDNA was synthesized using the SMARTScribe Reverse Transcriptase kit (Clontech). Briefly, 3 μg of total RNA was mixed with 1 μl of TB1/IgG1 primer (10 μM) in a sterile thin-walled PCR tube to a final volume of 4 μl (Mix 1). After centrifugation, Mix 1 was incubated at 70°C for 4 min, followed by 42°C for 2 min to anneal the priming oligo. The cDNA synthesis reaction (Mix 2) was prepared by combining 2 μl of 5X First Strand Buffer, 1 μl of DTT (20 mM), 1 μl of 5’-SA primer (10 μM), 1 μl of dNTP mix (10 mM), and 1 μl of SMARTScribe Reverse Transcriptase (100 U/μl) to a final volume of 6 μl. Mix 2 was added to Mix 1 for a final volume of 10 μl. The mixture was centrifuged briefly and incubated at 42°C for 60 min, followed by 70°C for 15 min.

##### First PCR amplification (PCR1)

The cDNA was amplified by PCR using PrimeSTAR GXL DNA Polymerase (Takara). The 50 μl PCR reaction contained 10 μl of 5X PrimeSTAR GXL Buffer, 4 μl of dNTP mix (2.5 mM), 2 μl of 5’S1 primer (10 μM), 2 μl of TB2/IgG2 primer (10 μM), 8 μl of cDNA, 1 μl of PrimeSTAR GXL DNA Polymerase, and 23 μl of nuclease-free water. Cycling conditions were: 98°C for 1 min; 21 cycles of 98°C for 10 s, 60°C for 15 s, and 68°C for 50 s; final extension at 68°C for 5 min. PCR products were purified using the DNA Clean-up Kit (CWBIO) and quantified by Qubit 4.0 fluorometry.

##### Second PCR amplification (PCR2)

The purified PCR1 products were further amplified by a second PCR using PrimeSTAR GXL DNA Polymerase (Takara). The 50 μl reaction contained 10 μl of 5X PrimeSTAR GXL Buffer, 4 μl of dNTP mix (2.5 mM), 2 μl of 5’S2 primer (10 μM), 2 μl of BCJ/IgGJ primer (10 μM), 10 μl of purified PCR1 product, 1 μl of PrimeSTAR GXL DNA Polymerase, and 21 μl of nuclease-free water. Cycling conditions were: 98°C for 1 min; 18 cycles of 98°C for 10 s, 60°C for 15 s, and 68°C for 50 s; final extension at 68°C for 5 min. PCR2 products were purified using the DNA Clean-up Kit (CWBIO) and quantified by Qubit fluorometry. Products were analyzed by agarose gel electrophoresis to verify library fragment sizes.

Primer sequences:

5′-SA: AAGCAGTGGTATCAACGCAGAGTACTCTTrGrG3; TB1: CAGTATCTGGAGTCATTGA; IgG1: GTGTTGCTGGGCTTGTG; 5′S1: AAGCAGTGGTATCAACGCAG; TB2: TGCTTCTGATGGCTCAAACAC; IgG2: GTGTTGCTGGGCTTGTG; 5′S2: AAGCAGTGGTATCAACGCAG; BCJ: ACACSTTKTTCAGGTCCTC; IgGJ: GAGGAGACGGTGACCRKGGT;.

##### Sequencing library construction and quality control

Sequencing libraries were constructed from 250 ng of purified PCR2 products using the TIANSeq Fast DNA Library Kit (TIANGEN) with TIANSeq Single-Indexed Adapters (Illumina). End repair, A-tailing, and adapter ligation were performed following the manufacturer’s protocol. Adapter-ligated products were purified with the DNA Clean-up Kit (CWBIO) and further amplified by PCR. Final libraries were purified by an additional DNA Clean-up Kit step and size-selected for 400-800 bp fragments using AMPure XP beads. Libraries were quantified by Qubit fluorometry and the insert size distribution was analyzed on an Agilent 2100 Bioanalyzer. Libraries with the expected insert size were accurately quantified by qRT-PCR to ensure the final concentration was greater than 2 nM.

### Data analysis

Raw sequence reads are processed to identify V, D, and J genes and complementarity determining region 3(CDR3) sequences using the MiXCR tool v3.0.9, reference germline V, D, J, and C gene sequences were downloaded from IMGT database (31, 32).

### TMB score calculating method

Germline variants were annotated using dbNSFP by software SnpEff and filtered with population frequency less than 0.001 (33, 34). All variants beyond the targeted exon region were removed. All remaining variants were annotated with annovar (35). The function somatic variant was the variant that can lead to changes in the coding protein. Nonfunction somatic variants were the opposite. Functional tumor mutation burden (TMB) and Total TMB were calculated. Total TMB = number of variants/size of targeted exon region. Functional TMB = number of functional somatic variants/size of targeted exon region.

### HLA alleles calling

HLA class I type of Patients were obtained through the software Optitypeby using WES reads (36).

### Tumor neoantigen burden (TNB) score calculating method

Considering that each HLA type I allele may have different expression intensities, we quantified the expression of each of the six HLA type I alleles corresponding to each patient separately. All transcripts involved in somatic mutations were designed with self-developed software to 8-11 amino acids (AA) peptides containing the mutant site. For frameshift mutations, all sequences from the start of the shift mutation to the stop codon were included, and to the end of the cDNA if there was no stop codon. The binding of all 8-11 AA peptides to all HLA type I alleles was predicted by netmhcpan4.0 (37). The binding scores of each 8-11 peptide to all HLA alleles were calculated. Peptides that intersect would merge into 25AA neoantigen, and the 25AA neoantigen score was equal to the sum of all peptide scores multiplied by the RNA mutation frequency at that site.

### Statistical methods

All statistical analyses were performed using R software (version 4.2.1, R Foundation for Statistical Computing, Vienna, Austria). Correlation analyses were performed using R, and p-value was calculated by cor.test() function.

## Results

### Mutational landscape of patients’ GBM tumors, HLA typing, and immune cell components of TME

**Figure 1:**
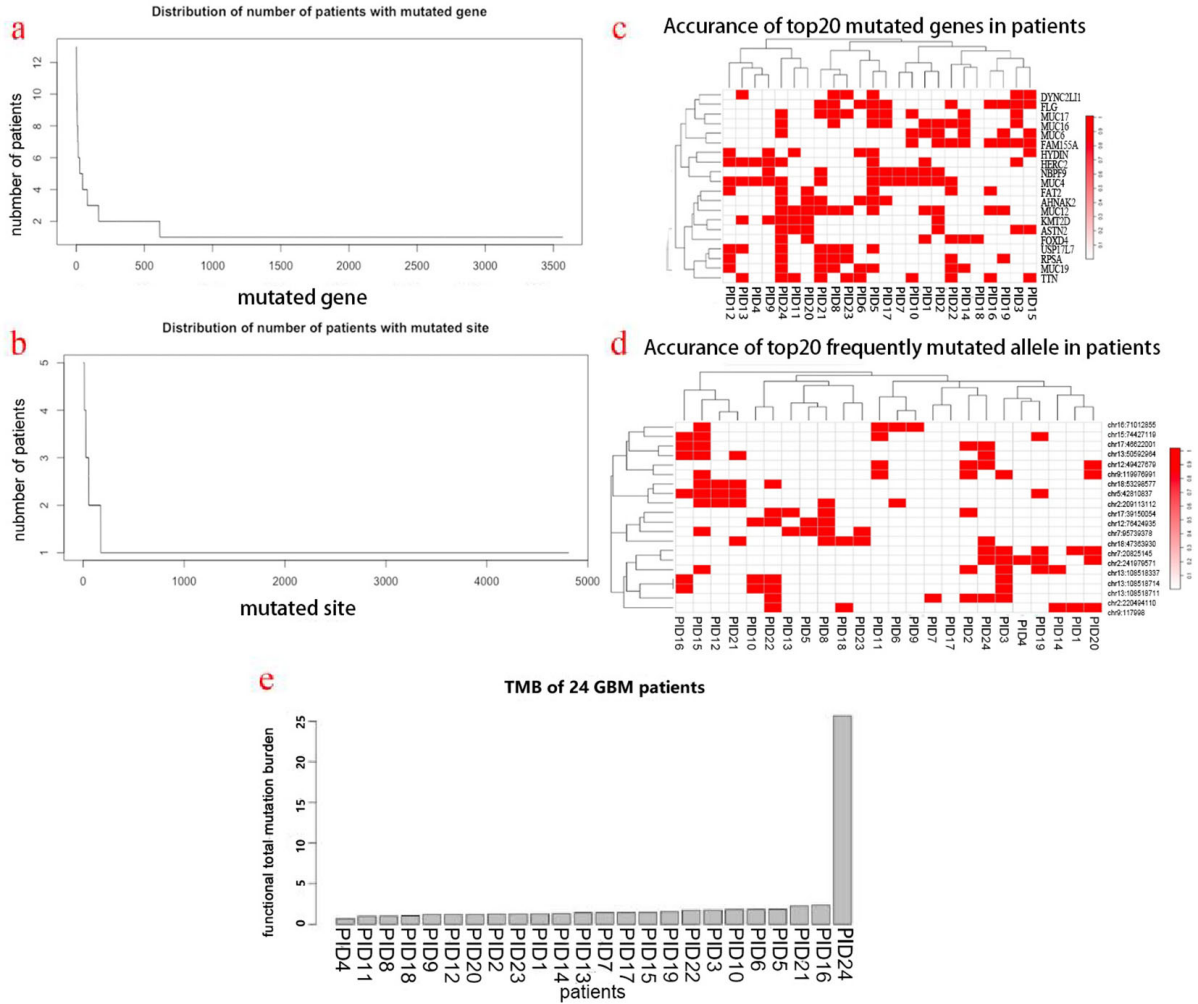
Mutational landscape of patients’ GBM tumors and HLA typing. **(a)** Distribution of the number of patients with mutated genes. **(b)** Distribution of the number of patients with mutated sites. **(c)** Top 20 most frequent mutated genes in patients. **(d)** Top 20 most frequent mutated sites in patients. **(e)** TMB distribution of patients.

In total, there were 3567 mutated genes involving 4810 mutated sites in these 24 GBM patients, averaging 148 mutated genes and 200 mutated sites per patient. The distribution of numbers of patients with mutated genes and sites are shown in **Figures 1A, B**. In general, most majority of mutated genes and sites occurred in only one patient, implying the inherent inter-tumor heterogeneity. However, there were multiple mutated genes and sites shared by more than one patient, and the top 20 most frequent mutated genes and sites were demonstrated in **Figures 1C, D**. Of note, the IDH 1 mutation appeared in five patients (21%). As a whole, the total mutation burden (TMB) of these patients was low (less than 5 Muts/MB), except for one patient bearing an extremely high mutation load (**Figure 1E**).

### HLA typing

**Figure 2:**
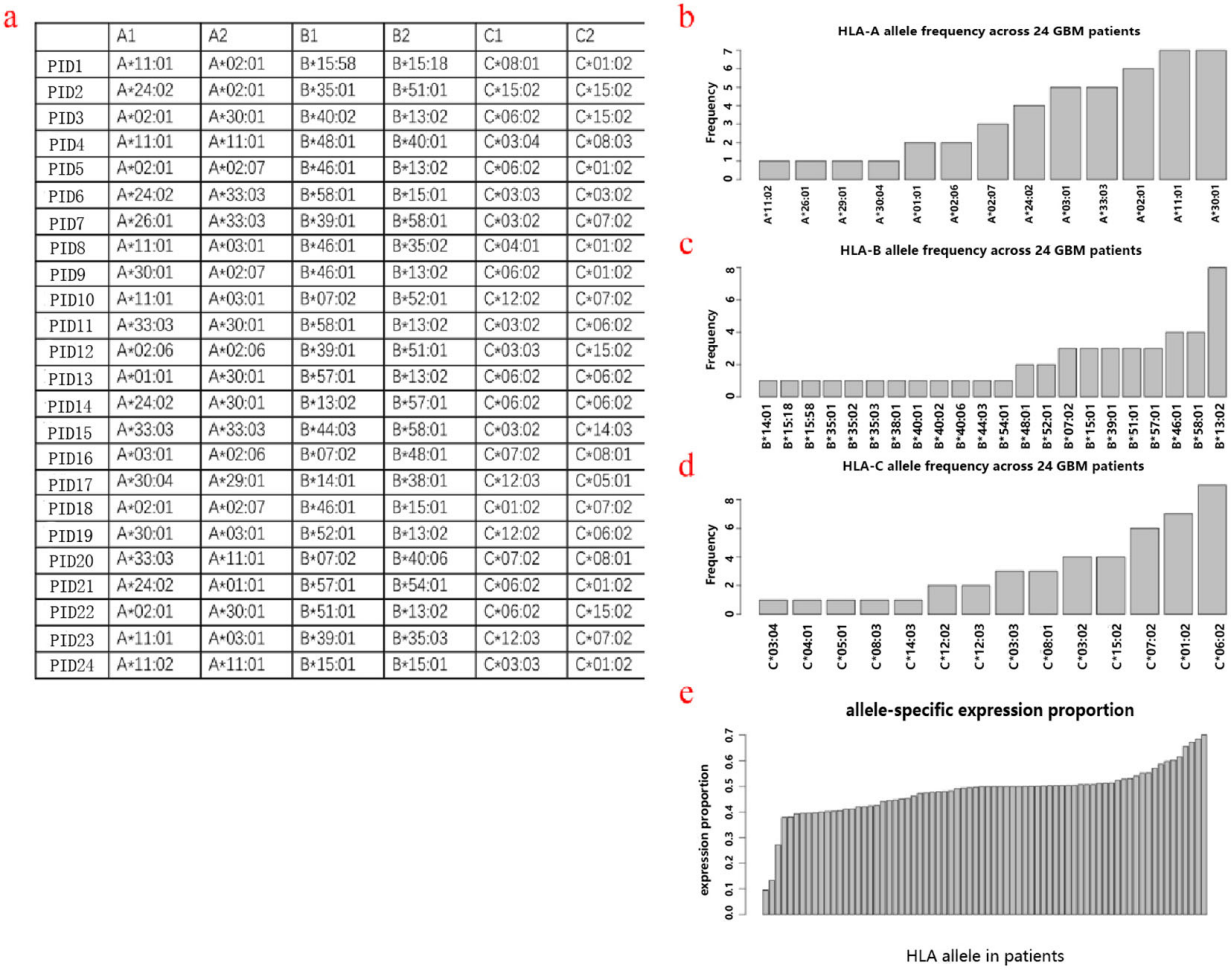
HLA typing. The overall HLA typing of these 24 patients **(a)**. HLA frequency across patients in alleles A **(b)**, B **(c)**, and C **(d)**. HLA allele-specific expression proportion **(e)**.

The Human Leukocyte Antigen (HLA) genes are critical components of the human immune system. They are essential for presenting neoantigens on the cell surface, thereby enabling their recognition by T cells. The HLA typing was determined by the application of OptiType algorithm, based on the data of WES&RNA sequencing of tumor tissue and peripheral white blood cells. The specific HLA typing of each patient was described (**Figure 2A**). Among these 24 patients, 17 have completely different genotypes, and 7 patients have the same genotype. The frequency of each genotype is shown in **Figures 2B–D**, with HLA-A30:01&HLA-A11:01, HLA-B13:02, and HLA-C06:02, as the most frequent genotypes at their respective allele sites. The expression proportion of each allele was determined, which demonstrated unequal expression of several allelic sites indicating heterogeneous penetration (**Figure 2E**).

### Immune cell components of TME

**Figure 3:**
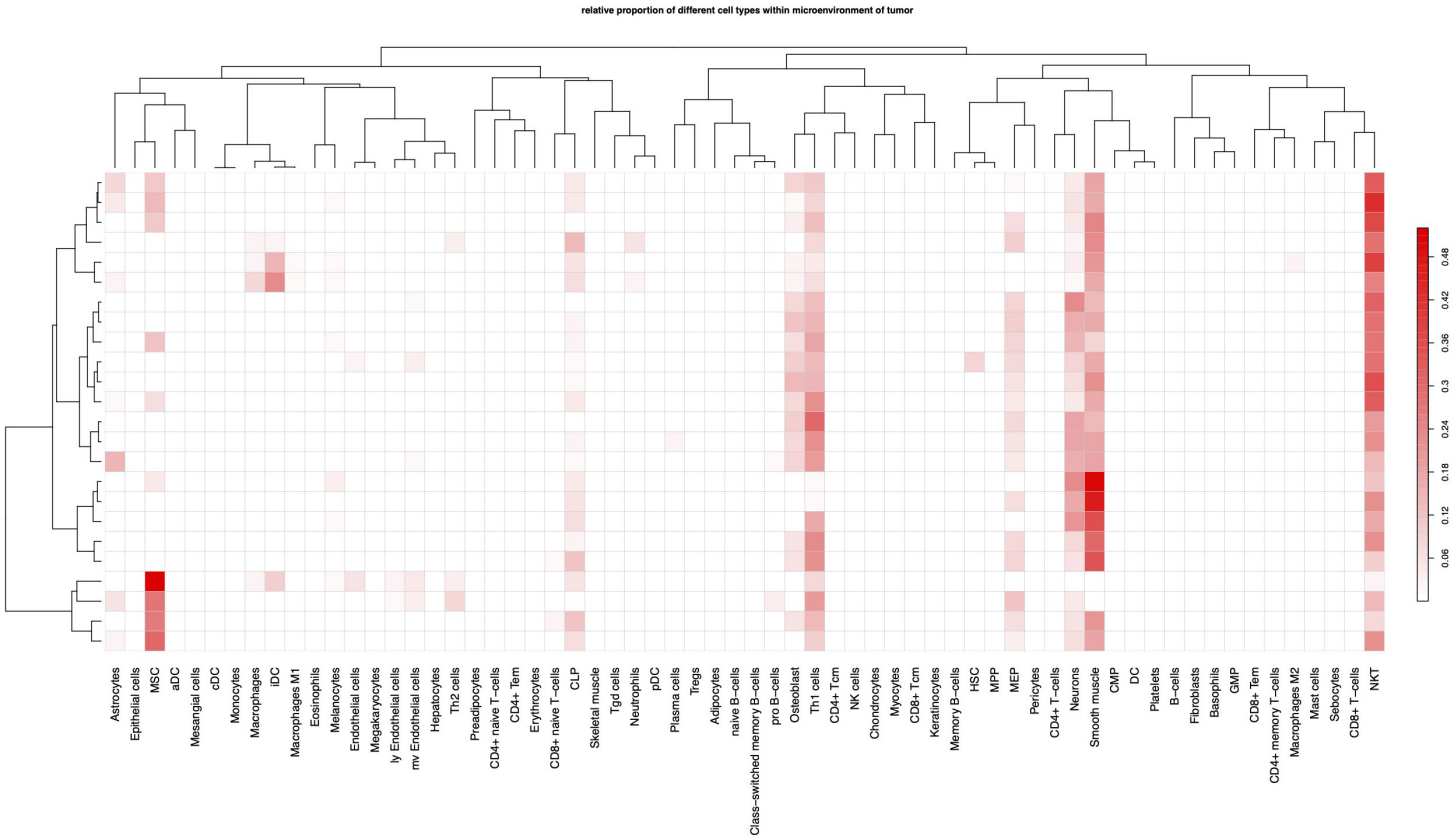
Immune cell components of TME. Each column indicates a type of cell, each row represents a patient.

Since the tumor TMB plays a critical role in the immune response, we analyzed the cellular components of these 24 GBM tumor tissues by bioinformatics. The proportion of each immune cell was calculated by xCell algorithm (38). The most abundant immune cells were NKT and Th1, while the other effector cells such as CD8+T, were minimal (**Figure 3**), indicating a “cold” immune TMB as previously reported.

### Profiling of TCR/BCR repertoire

**Figure 4:**
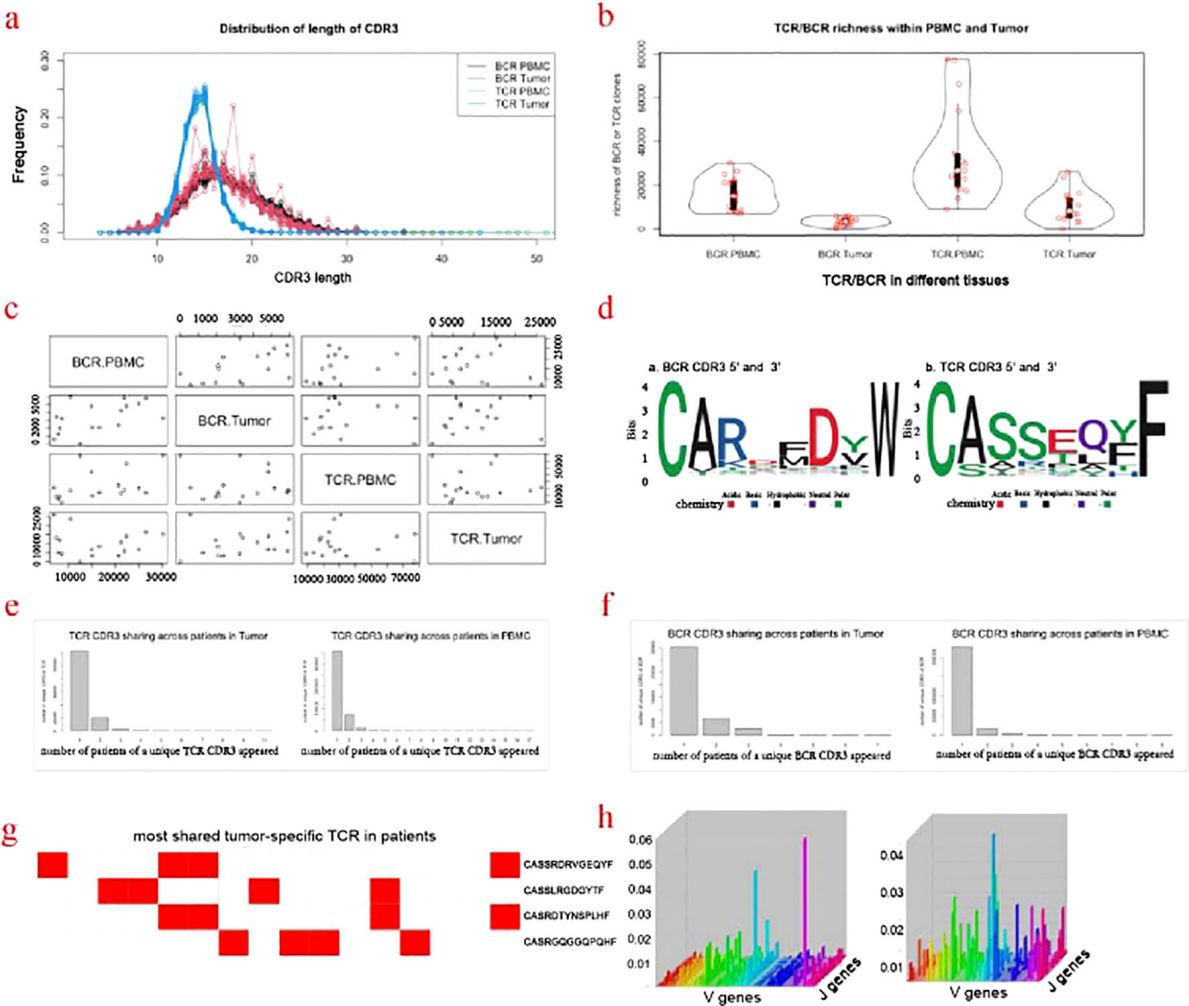
Profiling of TCR/BCR repertoire. **(a)** Distribution of length of CDR3. **(b)** TCR/BCR richness within PMBC and Tumor. **(c)** Correlation of the CDR3 of TCR and BCR between the tumor tissue and PBMC. **(d)** Motifs of the four AAs in the N- and C-terminal of CDR3 of TCR and BCR. **(e)** TCR CDR3 sharing across patients in Tumor and PMBC. **(f)** BCR CDR3 sharing across patients in Tumor and PMBC. **(g)** Most shared tumor-specific TCR CDR3 in more than 4 patients. **(h)** The re-arrangement of the TCR VJ genes in PBMC and tumor tissue.

TCR (T Cell Receptor) and BCR (B Cell Receptor) are key markers of cellular and humoral immunity, respectively. T cell receptors (TCR) are associated with cell-mediated immunity, while B cell receptors (BCR) are central to humoral immunity. Both immunity types are critical in the immune response against cancer and neoantigens, and could work collaboratively to recognize neoantigen in cancer cells. In this study, the repertoire of TCR and BCR including CDR3 regions of the tumor tissue and PBMC was profiled. In general, the distribution of the length of CDR3 of tumor tissue and PBMC were similar, both for TCR and BCR. The length of TCR CDR3 was shorter than that of BCR, while their peak values were similar (at 15 aa). Compared with the BCR CDR3 of the PBMC, the BCR CDR3 has another four peak values at 14, 18, 20, and 23 aa (**Figure 4A**). The richness of TCR/BCR was defined by the quantity of the clones/types. In both the tumor tissue and PMBC, the richness of TCR is higher than that of BCR (**Figure 4B**). The richness of BCR in tumor tissue was significantly correlated with that in PMBC (rr=0.5, p-value =0.039), while there is no notable correlation for TCR (rr=0.018, p-value=0.98). Interestingly, there is a remarkable correlation between TCR and BCR within tumor tissue (rr=0.59, p-value=0.017) (**Figure 4C**). The affinity of TCR or BCR largely depends on the amino acid composition of the CDR3 sequence. The N-terminal of these CDR3 sequences of TCR and BCR was predominately initiated by “CA” amino acids (AA). Other than this, there is variety across the CDR3 sequence. By comparing the four AAs of N-terminal and C-terminal between the CDR3 of TCR and BCR, there is more diversity seen in the CDR3 of BCR (**Figure 4D**). Most CDR3 sequences are unique for each patient. For TCR, 16.3% and 20% CDR3 occurred in more than two patients in tumor tissue and PMBC, respectively (**Figure 4E**). There were more shared CDR3 sequences of TCR in PMBC, compared with that in tumor tissue. CDR3 sequence “CASSLEETQYF” appeared in the PMBC of every patient. This sequence was reported to be associated with the infection of CMV and Tubercle Bacillus (39). For BCR, 21% and 8.7% CDR3 occurred in more than two patients in tumor tissue and PMBC, respectively (**Figure 4F**). Be different from TCR, there were more shared CDR3 sequences of BCR in tumor tissue, compared with that of PMBC. Furthermore, no single BCR CDR3 sequence was shared by every patient both in tumor tissue or PMBC. In total, 6,786 TCR CD3 sequences occurred in the tumor tissue but not in PMBC, in more than two patients, suggesting the tumor-specific. Among these, there were four TCR CDR tumor-specific sequences occurred in more than four patients (**Figure 4G**). Further analysis demonstrated a different rearrangement of VJ genes of TCR between tumor tissue and PMBC (**Figure 4H**).

### Identification of neoantigens

**Figure 5:**
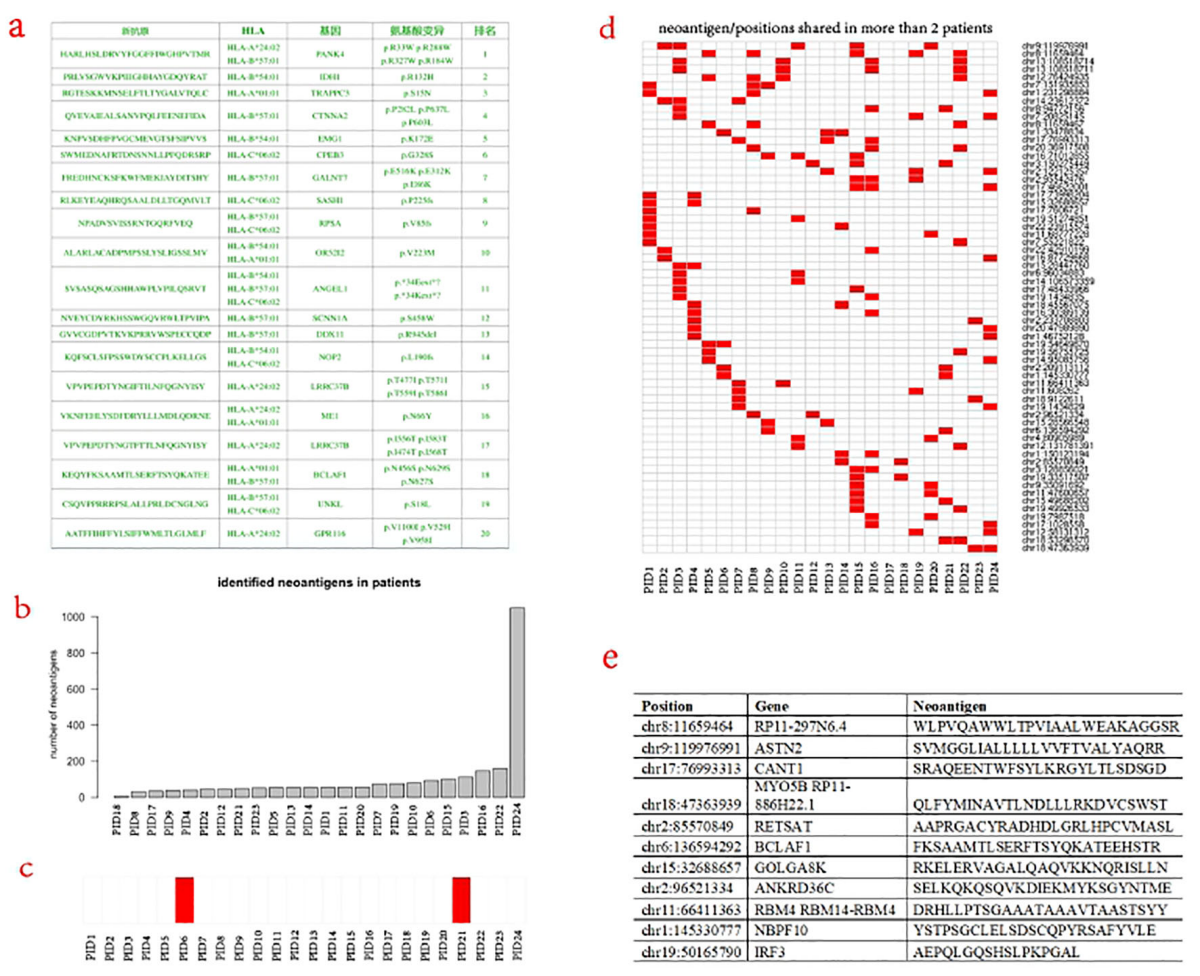
Identification and design of neoantigen vaccine. **(a)** The design of the top 20 neoantigens for one patient. **(b)** Distribution of the identified neoantigens across patients. **(c)** IDH1 associated neoantigen were identified in only two patients. **(d)** Neoantignes and sites shared in more than 2 patients. **(e)** Minimum of neoantigen bulk for the 24-patient cohort.

Based on the above data, it is feasible to calculate and design the neoantigen peptide sequence for each patient. The top twenty neoantigen peptide sequences of one patient were described (**Figure 5A**). Among these 24 patients, the mean number of neoantigen peptides was 107, with 23 patients with less than 200 and one patient with more than 1,000 (**Figure 5B**). As mentioned above, there were 5 patients with IDH1 mutation. After analysis, the mutated IDH1 could be presented by HLA as neoantigen in only 2 patients (**Figure 5C**). The most frequent neoantigens are located at the chr8:11659464 and chr9:119976991, corresponding to the RP11-297N6.4 and ASTN2 genes, respectively (**Figure 5D**). RP11-297N6.4 is a non-coding gene and recent studies indicate its association with the generation of neoantigens as genome “dark matter” (40). ASTN2 was reported as an important biomarker for GBM by the single-cell sequencing study (41). Overall, there is vast heterogeneity across these neoantigen peptides, and very few patients share more than two neoantigen peptides. To design the “common” neoantigen for these 24 patients, we apply the Entropy, Mutual Information, and Greedy Algorithm. It turned out that at least, an 11-peptide repertoire is required by the greedy algorithm to cover these 24 patients providing each patient with at least one neoantigen peptide (**Figure 5E**).

## Discussion

The prognosis of patients with GBM remains poor and recent breakthroughs in cancer therapy do not bring major changes for these patients (3). Therapeutic cancer vaccines, mostly immune cell-based, have gained approval for several types of human cancer (42). These proof-of-concept trials have dramatically boosted the enthusiasm for the development of more therapeutic vaccines with a wider anti-cancer spectrum. Cancer neoantigen is mostly caused by the accumulation of genomic alterations (16). Compared with tumor-associated-antigen (TAA), neoantigen has a major advantage in its specificity. The application of neoantigen, either as a cancer vaccine or in combination with other immune cells, has been tested in several clinical trials of human cancers including glioma (18, 19). The prerequisite for these applications is the identification of neoantigens.

To fully exploit the neoantigen repertoire of GBM, particularly in a Chinese GBM patients cohort, and also serve as a pilot study to our previously initiated clinical trial, we conducted this multi-omic profiling of GBM tumors and its immune TMB. There is vast heterogeneity across the mutations, HLA typing, and TCR/BCR repertoire. On average, 200 mutated sites occurred in 148 genes. Dr. Keskin and his colleague reported an average of 116 somatic single-nucleotide variants per tumor (range, 75–158) with a median of 59 coding mutations per tumor (19). This difference may be due to ethical genetic background. Nevertheless, this low mutation rate is consistent with previous studies. We did not identify any predominant mutated gene in this cohort. IDH1 mutation is a major event in lower-grade glioma, with a mutation rate ranging from 65-90%. For GBM, the alteration rate of the IDH1 gene is lesser, ranging from 3-7% (43). In our cohort, 5 (20%) have the mutated IDH1 gene, 2 (8%) of which could be predicted to have neoantigen. Dr. Keskin and his colleague did not identify IDH1 mutation in 10 GBM patients enrolled in their neoantigen trial (19). Extensive heterogeneity exists in the HLA typing, either in the genotype of the allele or in the expression rate of each allele. Previous studies did not detail the profiling of HLA typing of glioma. A few reports have shown the correlation of HLA typing with the incidence of glioma (44–47). None of the most frequent HLA genotypes in our cohort are involved in these correlations.

We revealed an abundance of NKT and Th1 cells, and scant existence of CD8+T cells, within the TME of GBM. Previous studies have demonstrated macrophage and microglial cells as the predominant immune cells within the TMC (48, 49). This difference could be due to the different races, molecular subtypes of glioma, and obtained regions of tumor tissue. The poor existence of CD8+T cells implies a lack of infiltration by cytotoxic T cells, which may contribute to the failure of checkpoint inhibitors. The role of NKT and Th1 cells with the TME deserve further studies. To the best of our knowledge, no study systemically analyzes the BCR and TCR of tumor tissue and PMBC in glioma. The cluster of CDR3 of TCR/BCR within the GBM tumor indicates an immune response within tumor tissue, which is further confirmed by the decreased richness/clone types of TCR/BCR within the tumor tissue. The broad CDR3 variations of TCR/BCR could not allow a consensus on a “general motif”, which reflected the immunogenic heterogeneity of tumor cells.

Very few neoantigens were shared by different patients. The top two most frequent neoantigens identified were located at chr8:11659464 and chr9:119976991, corresponding to the gene RP11-297N6.4 and ASTN2, respectively. Despite this vast immunogenic heterogeneity, it is feasible to design a therapeutic vaccine bulk for these patients as a cohort. A minimum of 11 peptide bulk is developed to warrant each patient has at least one neoantigen. It is likely that, with the increased number of enrolled patients, the number of the neoantigen peptides in this vaccine bulk will need to be expanded.

### Summary

In conclusion, vast heterogeneity exists across the neoantigens, HLA typing, and TCR/BCR repertoire of GBM tumors, which was accompanied by a “cold” immune TMB. Despite this heterogeneity, it is feasible to develop a therapeutic vaccine bulk to cover these patients as a cohort.

## Data Availability

The data presented in the study are deposited in the NGDC (National Genomics Data Center) repository, accession number HRA008820.

## References

[1] Ostrom QT Bauchet L Davis FG Deltour I Fisher JL Langer CE. The epidemiology of glioma in adults: a “state of the science” review. Neuro Oncol. (2014) 16:896–913. doi: 10.1093/neuonc/nou087 24842956 PMC4057143

[2] Ostrom QT Adel Fahmideh M Cote DJ Muskens IS Schraw JM Scheurer ME. Risk factors for childhood and adult primary brain tumors. Neuro Oncol. (2019) 21:1357–75. doi: 10.1093/neuonc/noz123 PMC6827837 31301133

[3] Wen PY Weller M Lee EQ Alexander BM Barnholtz-Sloan JS Barthel FP. Glioblastoma in adults: a Society for Neuro-Oncology (SNO) and European Society of Neuro-Oncology (EANO) consensus review on current management and future directions. Neuro Oncol. (2020) 22:1073–113. doi: 10.1093/neuonc/noaa106 PMC7594557 32328653

[4] Wu J Heidelberg RE Gajjar A. Adolescents and young adults with cancer: CNS tumors. J Clin Oncol. (2023) 42:686–95. doi: 10.1200/JCO.23.01747 PMC11550794 38064656

[5] Begagić E Pugonja R Bečulić H Čeliković A Tandir Lihić L Kadić Vukas S. Molecular targeted therapies in glioblastoma multiforme: A systematic overview of global trends and findings. Brain Sci. (2023) 13:1602. doi: 10.3390/brainsci13111602 38002561 PMC10669565

[6] Kaina BJ. Temozolomide, procarbazine and nitrosoureas in the therapy of Malignant gliomas: update of mechanisms, drug resistance and therapeutic implications. Clin Med. (2023) 12:7442. doi: 10.3390/jcm12237442 PMC10707404 38068493

[7] Cruz JVR Batista C Afonso BH Alexandre-Moreira MS Dubois LG Pontes B. Obstacles to glioblastoma treatment two decades after temozolomide. Cancers (Basel). (2022) 14:3203. doi: 10.3390/cancers14133203 35804976 PMC9265128

[8] Mellman I Coukos G Dranoff G. Cancer immunotherapy comes of age. Nature. (2011) 480:480–9. doi: 10.1038/nature10673 PMC3967235 22193102

[9] Topalian SL Taube JM Pardoll DM. Neoadjuvant checkpoint blockade for cancer immunotherapy. Science. (2020) 367:eaax0182. doi: 10.1126/science.aax0182 32001626 PMC7789854

[10] Sampson JH Gunn MD Fecci PE Ashley DM. Brain immunology and immunotherapy in brain tumours. Nat Rev Cancer. (2020) 20:12–25. doi: 10.1038/s41568-019-0224-7 31806885 PMC7327710

[11] Lin Q Ba T Ho J Chen D Cheng Y Wang L. First-in-human trial of ephA2-redirected CAR T-cells in patients with recurrent glioblastoma: A preliminary report of three cases at the starting dose. Front Oncol. (2021) 11:694941. doi: 10.3389/fonc.2021.694941 34235085 PMC8256846

[12] Vitanza NA Johnson AJ Wilson AL Brown C Yokoyama JK Künkele A. Locoregional infusion of HER2-specific CAR T cells in children and young adults with recurrent or refractory CNS tumors: an interim analysis. Nat Med. (2021) 27(9):1544–52. doi: 10.1038/s41591-021-01404-8 34253928

[13] Schalper KA Rodriguez-Ruiz ME Diez-Valle R López-Janeiro A Porciuncula A Idoate MA. Neoadjuvant nivolumab modifies the tumor immune microenvironment in resectable glioblastoma. Nat Med. (2019) 25:470–6. doi: 10.1038/s41591-018-0339-5 30742120

[14] O’Rourke DM Nasrallah MP Desai A Melenhorst JJ Mansfield K Morrissette JJD. A single dose of peripherally infused EGFRvIII-directed CAR T cells mediates antigen loss and induces adaptive resistance in patients with recurrent glioblastoma. Sci Transl Med. (2017) 9:eaaa0984. doi: 10.1126/scitranslmed.aaa0984 28724573 PMC5762203

[15] Ahmed N Brawley V Hegde M Bielamowicz K Kalra M Landi D. HER2-specific chimeric antigen receptor-modified virus-specific T cells for progressive glioblastoma: A phase 1 dose-escalation trial. JAMA Oncol. (2017) 3:1094–101. doi: 10.1001/jamaoncol.2017.0184 PMC5747970 28426845

[16] Peng M Mo Y Wang Y Wu P Zhang Y Xiong F. Neoantigen vaccine: an emerging tumor immunotherapy. Mol Cancer. (2019) 18:128. doi: 10.1186/s12943-019-1055-6 31443694 PMC6708248

[17] Ho SY Chang CM Liao HN Chou WH Guo CL Yen Y. Current trends in neoantigen-based cancer vaccines. Pharm (Basel). (2023) 16:392. doi: 10.3390/ph16030392 PMC10056833 36986491

[18] Platten M Bunse L Wick A Bunse T Le Cornet L Harting I. A vaccine targeting mutant IDH1 in newly diagnosed glioma. Nature. (2021) 592:463–8. doi: 10.1038/s41586-021-03363-z PMC8046668 33762734

[19] Keskin DB Anandappa AJ Sun J Tirosh I Mathewson ND Li S. Neoantigen vaccine generates intratumoral T cell responses in phase Ib glioblastoma trial. Nature. (2019) 565:234–9. doi: 10.1038/s41586-018-0792-9 PMC6546179 30568305

[20] Mueller S Taitt JM Villanueva-Meyer JE Bonner ER Nejo T Lulla RR. Mass cytometry detects H3. 3K27M-specific Vaccine responses diffuse midline glioma. J Clin Invest. (2020) 130:6325–37. doi: 10.1172/JCI140378 PMC7685729 32817593

[21] Katsikis PD Ishii KJ Schliehe C. Challenges in developing personalized neoantigen cancer vaccines. Nat Rev Immunol. (2023) 24:213–27. doi: 10.1038/s41577-023-00937-y PMC12001822 37783860

[22] Rafii S Kandoussi S Ghouzlani A Naji O Reddy KP Ullah Sadiqi R. Deciphering immune microenvironment and cell evasion mechanisms in human gliomas. Front Oncol. (2023) 13:1135430. doi: 10.3389/fonc.2023.1135430 37274252 PMC10235598

[23] Bolger AM Lohse M Usadel B. Trimmomatic: a flexible trimmer for Illumina sequence data. Bioinformatics. (2014) 30:2114–20. doi: 10.1093/bioinformatics/btu170 PMC4103590 24695404

[24] de Sena Brandine G Smith AD. Falco: high-speed FastQC emulation for quality control of sequencing data. F1000Res. (2019) 8:1874. doi: 10.12688/f1000research 33552473 PMC7845152

[25] Li H Durbin R. Fast and accurate short read alignment with Burrows-Wheeler transform. Bioinformatics. (2009) 25:1754–60. doi: 10.1093/bioinformatics/btp324 PMC2705234 19451168

[26] Okonechnikov K Conesa A García-Alcalde F. Qualimap 2: advanced multi-sample quality control for high-throughput sequencing data. Bioinformatics. (2016) 32:292–4. doi: 10.1093/bioinformatics/btv566 PMC4708105 26428292

[27] Van der Auwera GA Carneiro MO Hartl C Poplin R Del Angel G Levy-Moonshine A. From FastQ data to high confidence variant calls: the Genome Analysis Toolkit best practices pipeline. Curr Protoc Bioinf. (2013) 43:11.10.1–11.10.33. doi: 10.1002/0471250953.2013.43.issue-1 PMC4243306 25431634

[28] Mayakonda A Lin DC Assenov Y Plass C Koeffler HP. Maftools: efficient and comprehensive analysis of somatic variants in cancer. Genome Res. (2018) 28:1747–56. doi: 10.1101/gr.239244.118 PMC6211645 30341162

[29] Kim D Paggi JM Park C Bennett C Salzberg SL. Graph-based genome alignment and genotyping with HISAT2 and HISAT-genotype. Nat Biotechnol. (2019) 37:907–15. doi: 10.1038/s41587-019-0201-4 PMC7605509 31375807

[30] Pertea M Pertea GM Antonescu CM Chang TC Mendell JT Salzberg SL. StringTie enables improved reconstruction of a transcriptome from RNA-seq reads. Nat Biotechnol. (2015) 33:290–5. doi: 10.1038/nbt.3122 PMC4643835 25690850

[31] Bolotin DA Poslavsky S Mitrophanov I Shugay M Mamedov IZ Putintseva EV. MiXCR: software for comprehensive adaptive immunity profiling. Nat Methods. (2015) 12:380–1. doi: 10.1038/nmeth.3364 25924071

[32] Lefranc MP. IMGT, the international ImMunoGeneTics database. Nucleic Acids Res. (2001) 29:207–9. doi: 10.1093/nar/29.1.207 PMC29797 11125093

[33] Liu X Wu C Li C Boerwinkle E. dbNSFP v3.0: A one-stop database of functional predictions and annotations for human nonsynonymous and splice-site SNVs. Hum Mutat. (2016) 37:235–41. doi: 10.1002/humu.2016.37.issue-3 PMC4752381 26555599

[34] Cingolani P Platts A Wang le L Coon M Nguyen T Wang L. A program for annotating and predicting the effects of single nucleotide polymorphisms, SnpEff: SNPs in the genome of Drosophila melanogaster strain w1118; iso-2; iso-3. Fly (Austin). (2012) 6:80–92. doi: 10.4161/fly.19695 22728672 PMC3679285

[35] Wang K Li M Hakonarson H. ANNOVAR: functional annotation of genetic variants from high-throughput sequencing data. Nucleic Acids Res. (2010) 38:e164. doi: 10.1093/nar/gkq603 20601685 PMC2938201

[36] Szolek A Schubert B Mohr C Sturm M Feldhahn M Kohlbacher O. OptiType: precision HLA typing from next-generation sequencing data. Bioinformatics. (2014) 30:3310–6. doi: 10.1093/bioinformatics/btu548 PMC4441069 25143287

[37] Jurtz V Paul S Andreatta M Marcatili P Peters B Nielsen MJ. NetMHCpan-4.0: improved peptide-MHC class I interaction predictions integrating eluted ligand and peptide binding affinity data. Immunol. (2017) 199:3360–8. doi: 10.4049/jimmunol.1700893 PMC5679736 28978689

[38] Aran D Hu Z Butte AJ. xCell: digitally portraying the tissue cellular heterogeneity landscape. Genome Biol. (2017) 18:220. doi: 10.1186/s13059-017-1349-1 29141660 PMC5688663

[39] Chu ND Bi HS Emerson RO Sherwood AM Birnbaum ME Robins HS. Longitudinal immunosequencing in healthy people reveals persistent T cell receptors rich in highly public receptors. BMC Immunol. (2019) 20:19. doi: 10.1186/s12865-019-0300-5 31226930 PMC6588944

[40] Capietto AH Hoshyar R Delamarre L. Sources of cancer neoantigens beyond single-nucleotide variants. Int J Mol Sci. (2022) 23:10131. doi: 10.3390/ijms231710131 36077528 PMC9455963

[41] Guo T Bao A Xie Y Qiu J Piao H. Single-cell sequencing analysis identified ASTN2 as a migration biomarker in adult glioblastoma. Brain Sci. (2022) 12:1472. doi: 10.3390/brainsci12111472 36358398 PMC9688571

[42] Saxena M van der Burg SH Melief CJM Bhardwaj N. Therapeutic cancer vaccines. Nat Rev Cancer. (2021) 21:360–78. doi: 10.1038/s41568-021-00346-0 33907315

[43] Sun H Yin L Li S Han S Song G Liu N. Prognostic significance of IDH mutation in adult low-grade gliomas: a meta-analysis. J Neurooncol. (2013) 113:277–84. doi: 10.1007/s11060-013-1107-5 23504258

[44] Choi SS Choi H Baek IC Park SA Park JS Kim TG. HLA polymorphisms and risk of glioblastoma in Koreans. PloS One. (2021) 16:e0260618. doi: 10.1371/journal.pone.0260618 34882724 PMC8659341

[45] Han S Deng J Wang Z Liu H Cheng W Wu A. Decreased human leukocyte antigen A*02:01 frequency is associated with risk of glioma and existence of human cytomegalovirus: a case-control study in Northern China. Cancer Immunol Immunother. (2017) 66:1265–73. doi: 10.1007/s00262-017-2018-7 PMC11028914 28523518

[46] Guerini FR Agliardi C Zanzottera M Delbue S Pagani E Tinelli C. Human leukocyte antigen distribution analysis in North Italian brain Glioma patients: an association with HLA-DRB1*14. J Neurooncol. (2006) 77:213–7. doi: 10.1007/s11060-005-9032-x 16314951

[47] Machulla HK Steinborn F Schaaf A Heidecke V Rainov NG. Brain glioma and human leukocyte antigens (HLA)–is there an association. J Neurooncol. (2001) 52:253–61. doi: 10.1023/A:1010612327647 11519856

[48] Fu W Wang W Li H Jiao Y Huo R. Single-cell atlas reveals complexity of the immunosuppressive microenvironment of initial and recurrent glioblastoma. Front Immunol. (2020) 11:835. doi: 10.3389/fimmu.2020.00835 32457755 PMC7221162

[49] Darmanis S Sloan SA Croote D Mignardi M Chernikova S Samghababi P. Single-cell RNA-seq analysis of infiltrating neoplastic cells at the migrating front of human glioblastoma. Cell Rep. (2017) 21:1399–410. doi: 10.1016/j.celrep.2017.10.030 PMC5810554 29091775

