# Supplementary material for: Integrative multi-omic profiling of the neoantigen landscape of glioblastoma for the development of therapeutic vaccines reveals vast heterogeneity in immunogenic signatures
Source: Front Oncol. 2025 Mar 21;15:1507632. doi: 10.3389/fonc.2025.1507632 (PMC11968714; doi:10.3389/fonc.2025.1507632)
Supplement: Supplementary file 1 [file SupplementaryFile1.docx]

**Supplementary materials**

1. ***Immune repertoire***

**1.1 Sample Collection and Preparation**

RNA was extracted using RNA Easy Fast Tissue/Cell Kit（TIANGEN） kit following the manufacturer's protocol. RNA purity was assessed by spectrophotometry (OD260/280 and OD260/230 ratios) using a NanoDrop ultra-micro spectrophotometer.

**1.2 Library Construction and Quality Control**

cDNA Synthesis

cDNA was synthesized using the SMARTScribe Reverse Transcriptase kit (Clontech). Briefly, 3 μg of total RNA was mixed with 1 μl of TB1/IgG1 primer (10 μM) in a sterile thin-walled PCR tube to a final volume of 4 μl (Mix 1). After centrifugation, Mix 1 was incubated at 70°C for 4 min, followed by 42°C for 2 min to anneal the priming oligo. The cDNA synthesis reaction (Mix 2) was prepared by combining 2 μl of 5X First Strand Buffer, 1 μl of DTT (20 mM), 1 μl of 5’-SA primer (10 μM), 1 μl of dNTP mix (10 mM), and 1 μl of SMARTScribe Reverse Transcriptase (100 U/μl) to a final volume of 6 μl. Mix 2 was added to Mix 1 for a final volume of 10 μl. The mixture was centrifuged briefly and incubated at 42°C for 60 min, followed by 70°C for 15 min.

First PCR Amplification (PCR1)

The cDNA was amplified by PCR using PrimeSTAR GXL DNA Polymerase (Takara). The 50 μl PCR reaction contained 10 μl of 5X PrimeSTAR GXL Buffer, 4 μl of dNTP mix (2.5 mM), 2 μl of 5’S1 primer (10 μM), 2 μl of TB2/IgG2 primer (10 μM), 8 μl of cDNA, 1 μl of PrimeSTAR GXL DNA Polymerase, and 23 μl of nuclease-free water. Cycling conditions were: 98°C for 1 min; 21 cycles of 98°C for 10 s, 60°C for 15 s, and 68°C for 50 s; final extension at 68°C for 5 min. PCR products were purified using the DNA Clean-up Kit (CWBIO) and quantified by Qubit 4.0 fluorometry.

Second PCR Amplification (PCR2)

The purified PCR1 products were further amplified by a second PCR using PrimeSTAR GXL DNA Polymerase (Takara). The 50 μl reaction contained 10 μl of 5X PrimeSTAR GXL Buffer, 4 μl of dNTP mix (2.5 mM), 2 μl of 5’S2 primer (10 μM), 2 μl of BCJ/IgGJ primer (10 μM), 10 μl of purified PCR1 product, 1 μl of PrimeSTAR GXL DNA Polymerase, and 21 μl of nuclease-free water. Cycling conditions were: 98°C for 1 min; 18 cycles of 98°C for 10 s, 60°C for 15 s, and 68°C for 50 s; final extension at 68°C for 5 min. PCR2 products were purified using the DNA Clean-up Kit (CWBIO) and quantified by Qubit fluorometry. Products were analyzed by agarose gel electrophoresis to verify library fragment sizes.

**1.3 Primer sequences:**

5′-SA: AAGCAGTGGTATCAACGCAGAGTACTCTTrGrGrG

TB1: CAGTATCTGGAGTCATTGA

IgG1: GTGTTGCTGGGCTTGTG

5′S1: CACTCTATCCGACAAGCAGTGGTATCAACGCAG

TB2: TGCTTCTGATGGCTCAAACAC

IgG2: GAAGTAGTCCTTGACCAGGCA

5′S2: CACTCTATCCGACAAGCAGT

BCJ: ACACSTTKTTCAGGTCCTC

IgGJ: GAGGAGACGGTGACCRKGGT

**1.4 Sequencing**

Sequencing libraries were constructed from 250 ng of purified PCR2 products using the TIANSeq Fast DNA Library Kit (TIANGEN) with TIANSeq Single-Indexed Adapters (Illumina). End repair, A-tailing, and adapter ligation were performed following the manufacturer's protocol. Adapter-ligated products were purified with the DNA Clean-up Kit (CWBIO) and further amplified by PCR. Final libraries were purified by an additional DNA Clean-up Kit step and size-selected for 400-800 bp fragments using AMPure XP beads.

Libraries were quantified by Qubit fluorometry and the insert size distribution was analyzed on an Agilent 2100 Bioanalyzer. Libraries with the expected insert size were accurately quantified by qRT-PCR to ensure the final concentration was greater than 2 nM.

1. ***mRNA-seq***

**2.1 Sample Collection and Preparation**

Total RNA was extracted from samples using TRIzol reagent according to the manufacturer's instructions. RNA degradation and contamination were monitored by agarose gel electrophoresis. RNA purity was assessed by spectrophotometry using a NanoPhotometer spectrophotometer (IMPLEN). RNA integrity was evaluated using an Agilent 2100 Bioanalyzer (Agilent Technologies).

**2.2 Library Preparation for Transcriptome Sequencing**

Sequencing libraries were prepared from 1 μg of total RNA per sample using the NEBNext Ultra RNA Library Prep Kit for Illumina (NEB) following the manufacturer's protocol. Briefly, mRNA was enriched using oligo-dT magnetic beads. Fragmentation was carried out in NEBNext First Strand Synthesis Reaction Buffer. First strand cDNA synthesis was performed using random hexamer primers and M-MuLV Reverse Transcriptase (RNase H-). Second strand cDNA was subsequently synthesized using DNA Polymerase I and RNase H. Overhangs were converted into blunt ends and adenylated at 3’ ends. NEBNext adaptors with hairpin loop structures were ligated for hybridization. cDNA fragments of 250-300 bp were size-selected using AMPure XP beads (Beckman Coulter). Before PCR amplification, 3 μl of USER enzyme (NEB) was added to enzymatically digest the uracil-containing adaptors. PCR was performed using Phusion High-Fidelity DNA polymerase, Universal PCR primers and index primers. AMPure XP beads were used to purify the final PCR products. Library quality was assessed on an Agilent 2100 Bioanalyzer.

Index-coded libraries were clustered on a cBot Cluster Generation System using a TruSeq PE Cluster Kit v3-cBot-HS (Illumina). Clustered libraries were then sequenced on an Illumina Novaseq platform to generate 150 bp paired-end reads.

**2.3 Data pre-processing**

Raw sequencing reads in fastq format were processed using in-house Perl scripts to remove adapter sequences, poly-N-containing reads, and low-quality reads. The processed clean reads were used for subsequent analyses. Quality control metrics including Q20, Q30, and GC content were calculated for the clean data.

1. ***WES sequencing***

**3.1 Sample Evaluation**

DNA quality was assessed by agarose gel electrophoresis to check for degradation and potential contamination by RNA or proteins. DNA concentrations were quantified precisely using the Qubit fluorometer. Only DNA samples with concentrations ≥0.6 μg were used for library construction.

**3.2 Library Construction and Capture**

Sequencing libraries were constructed using the Agilent SureSelect Human All Exon V6 kit following the manufacturer's protocol. Genomic DNA was fragmented to 180-280 bp using a Covarissonicator. End repair and A-tailing were performed on the fragmented DNA before ligation to indexed sequencing adapters. The adapter-ligated libraries were pooled and hybridized to biotinylated probes targeting exonic regions. Streptavidin-coated magnetic beads were used to capture probe-bound fragments. Captured exons were amplified by PCR and the final libraries were evaluated for quality control.

**3.3 Library Quality Control**

Initial library quantification was done with Qubit 2.0 fluorometry. Insert sizes were validated on an Agilent 2100 Bioanalyzer to meet target range specifications. Final precise library quantification was performed by qPCR to ensure concentrations ≥3 nM for adequate cluster densities during sequencing.

**3.4 Sequencing**

Qualified libraries were sequenced on an Illumina NovaSeq 6000 platform to generate 150 bp paired-end reads based on the effective concentrations and desired data yield.
